# Acupuncture for chronic constipation in patients with diabetes mellitus: A protocol for systematic review

**DOI:** 10.1097/MD.0000000000024886

**Published:** 2021-03-05

**Authors:** Sufen Cui, Qiu Yang, Suhua Xie, Qian Liu, Guangju Zhou

**Affiliations:** The Affiliated Hospital of North Sichuan Medical College, Sichuan, China.

**Keywords:** acupuncture, chronic constipation, diabetes mellitus, systematic review

## Abstract

**Background:**

Assessing the effectiveness and safety of acupuncture for chronic constipation in patients with diabetes mellitus is the main purpose of this systematic review protocol.

**Methods:**

The following electronic databases will be searched from their respective inception dates to December 1st 2020: PubMed, the Cochrane Library, Embase, World Science Net, the Allied and Complementary Medicine Database, the Web of Science, China National Knowledge Infrastructure, the Chongqing VIP Chinese Science and Technology Periodical Database and the Wanfang Database. All published randomized controlled trials in English or Chinese related to acupuncture for constipation in patient with diabetes mellitus will be included. The Bristol stool scale, spontaneous complete bowel movements, and observing symptoms (yes/no) including defecation feeling, defecation weakness, feeling of incomplete evacuation, bloating, and flatulence were considered as primary measures. The treatment efficiency consideration according to Bristol stool scale was considered as secondary measure. Two reviewers will conduct the study selection, data extraction and assessment independently. The assessment of risk of bias and data synthesis will be conducted with Review Manager Software (RevMan) V.5.2.

**Results:**

The results will provide a high-quality synthesis of current evidence for researchers in this subject area.

**Conclusion:**

The conclusion of our study will provide an evidence to judge whether.

Acupuncture is an effective intervention for chronic constipation in patients with diabetes mellitus.

**Ethics and dissemination:**

Formal ethical approval is not necessary as the data cannot be individualized. The results of this protocol will be disseminated in a peer-reviewed journal or presented at relevant conferences.

**PROSPERO registration number:**

INPLASY202110079.

## 1 Introduction

Diabetes mellitus has been a widespread disease in both developed and developing countries.^[1]^ Patients with diabetes mellitus often suffer from several gastrointestinal symptoms such as diarrhea, chronic constipation and fecal incontinence,^[2]^ of which chronic constipation is most popular.^[3]^ Poor dietary habits, less fluid intake and low physical activity are significant factors leading to chronic constipation.^[4]^ Patients with chronic constipation caused by diabetes mellitus are suffering from impaired general health, social functioning and mental health.^[5]^ It is necessary to pay attention to this complication with its increasing prevalence.

The treatment available now include nonpharmacological treatment, food and dietary changes, exercise and lifestyle changes and medical management.^[4]^ However, these solutions are not so satisfying and have side effects.^[6,7]^

A randomized trial supports the use of acupuncture for chronic severe functional constipation,^[8]^ and this review will provide a high-quality synthesis of current evidence for researchers in this subject area.

## 2 Methods and analysis

### 2.1 Study registration

This systematic review protocol was registered with PROSPERO 2020 (registration number: INPLASY202110079). And the protocol report is in the base of the Preferred Reporting Items for Systematic Reviews and Meta-Analyses Protocols (PRISMA-P) declaration guidelines.^[9]^ The review will be performed in line with the PRISMA-P declaration guidelines.^[10]^

### 2.2 Inclusion criteria for study selection

#### 2.2.1 Type of study

RCTs of acupuncture therapy for chronic constipation in patients with diabetes mellitus without restrictions on publication status will be eligible for inclusion.

#### 2.2.2 Type of participant

Participants who were 18 years or older with chronic constipation caused by diabetes mellitus will be included in spite of the gender, race, education or economic status.

#### 2.2.3 Type of intervention

Acupuncture therapy, which includes manual acupuncture, body acupuncture, electroacupuncture, plum blossom needle, warm needling, and fire needling. Other methods, which includes transcutaneous electrical nerve stimulation, laser acupuncture, dry needling, cupping, and moxibustion will be excluded.

Comparison interventions, including sham acupuncture (including sham acupuncture at selected acupoints, sham acupuncture at non-acupoints, pseudo-acupuncture interventions, needling at inappropriate acupoints and nonpenetrating sham acupuncture), placebo, usual care, medication, no treatment, and other conventional therapies, will be included.^[11]^ In addition, the review of trials evaluating acupuncture combined with another treatment compared with other typical treatments alone will be included.

#### 2.2.4 Type of outcome measure

The Bristol stool scale, spontaneous complete bowel movements, and observing symptoms (yes/no) including defecation feeling, defecation weakness, feeling of incomplete evacuation, bloating, and flatulence were considered as primary measures. The treatment efficiency consideration according to Bristol stool scale was considered as secondary measure.^[12]^

### 2.3 Search methods for identification of studies

#### 2.3.1 Electronic data sources

The following electronic databases will be searched from their respective inception dates to 1st December 2020: PubMed, the Cochrane Library, Embase, World Science Net, the Allied and Complementary Medicine Database, the Web of Science, China National Knowledge Infrastructure, the Chongqing VIP Chinese Science and Technology Periodical Database and the Wanfang Database. All published randomized controlled trials in English or Chinese related to acupuncture for constipation in patient with diabetes mellitus will be included.

#### 2.3.2 Searching other resources

The reference lists of potentially missing eligible studies will be scanned ant the relevant conference proceedings will be scanned as well.

### 2.4 Search strategy

The search strategy for PubMed is shown in Table 1. The following search keywords will be used: acupuncture (e.g., “acupuncture” or “acupuncture therapy” or “body acupuncture” or “manual acupuncture” or “electroacupuncture” or “fire needling” or “plum blossom needling”; constipation (e.g., “Dyschezia” or “Colonic Inertia”); diabetes mellitus (e.g., “diabetes insidious” or “diet, diabetic” or “prediabetic state” or “scleroderma adultorum” or “glycation end products, advanced” or “glucose intolerance” or “gastroparesis”); randomized controlled trial (e.g., “randomized controlled trial” or “controlled clinical trial” or “random allocation” or “randomized” or “randomly” or “double-blind method” or “single-blind method” or “clinical trial”. The equivalent search keywords will be used in the Chinese databases.

**Table 1:** Search strategy for the Pubmed database.

Number	Search items
1	constipation. Mesh.
2	constipation. ti, ab
3	Colonic Inertia. ti, ab
4	1 or 2–3
5	diabetes mellitus. Mesh.
6	diabetes mellitus. ti, ab
7	diabetes insidious
8	diet, diabetic
9	prediabetic state
10	scleroderma adultorum
11	glycation end products, advanced
12	glucose intolerance
13	gastroparesis
14	5 or 6–13
15	Acupuncture. Mesh.
16	Acupuncture. ti, ab
17	Acupuncture therapy. Mesh.
18	Acupuncture therapy. ti, ab
19	(acupuncture) and (therapy). ti, ab
20	Acupuncture^∗^
21	Body acupuncture. ti, ab
22	(body) and (acupuncture). ti, ab
23	Manual acupuncture. ti, ab
24	(manual) and (acupuncture). ti, ab
25	Electroacupuncture. ti, ab
26	(electro) and (acupuncture). ti, ab
27	Fire needling. ti, ab
28	(fire) and (needling). ti, ab
29	Plum blossom needling. ti, ab
30	(plum) and (blossom) and (needling). ti, ab
31	15 or 16–30
32	randomized controlled trial. pt
33	controlled clinical trial. pt
34	randomized controlled trials. Mesh.
35	random allocation. Mesh.
36	randomized. ti, ab
37	randomly. ti, ab
38	double-blind method. Mesh
39	single-blind method. Mesh
40	clinical trial. pt
41	32 or 33–40
42	4 and 14 and 31 and 41

### 2.5 Data collection and analysis

#### 2.5.1 Selection of studies

**Figure 1:**
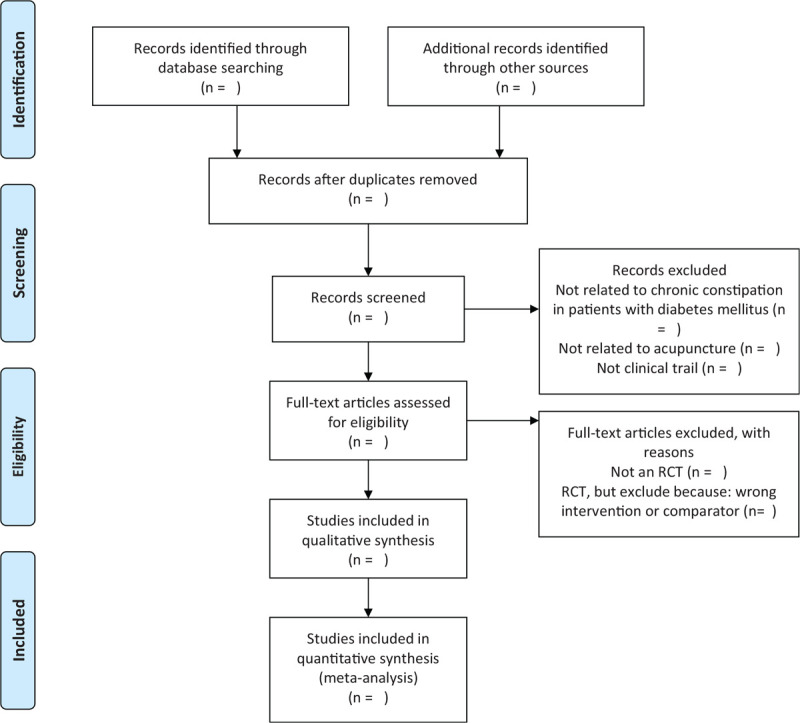
The PRISMA-P flow chart of the study selection process.

The titles and abstracts of all searched studies will be reviewed and screened independently by 2 reviewers, aiming at identifying eligible trials and eliminating duplicated or irrelevant studies in line with the criteria; the full text of all possibly eligible studies will obtained if necessary. A discussion with the third reviewer is planned to solve the disagreements. A PRISMA-P flow diagram will be used to show the study selection process (Fig. 1).

#### 2.5.2 Data extraction and management

The following data will be extracted from the selected studies by 2 independent reviewers using a standard data extraction sheet: year of publication, country, general information, participant characteristics, inclusion and exclusion criteria, sample size, randomization, blinding methods, methods, control, outcome measures, results, adverse reactions, conflicts of interest, ethical approval, and other information.

#### 2.5.3 Assessment of risk of bias and reporting of study quality

Two independent reviewers will access the quality of included literature and complete the Standards for Reporting Interventions in Clinical Trials of Acupuncture checklist with the Cochrane collaboration risk-of-bias assessment method.^[13]^

#### 2.5.4 Measures of treatment effect

Dichotomous data will be presented as risk ratio (RR) and 95 % confidence intervals, while continuous outcomes will be showed as standard mean difference (SMD) 95% CI.

#### 2.5.5 Unit of analysis issues

The individual participant will the analytical unit.

#### 2.5.6 Management of missing data

The cause of the missing data will be determined to solve the problem. And if this is not working, the authors will be contacted for the missing part. This will be documented and the available data will be extracted and analyzed if the missing data cannot be obtained.

#### 2.5.7 Assessment of heterogeneity

*I*^2^ test will be used to quantified inconsistency and standard χ^2^ test will be used to detect statistical heterogeneity. Studies will be considered to have homogeneity if the *P* value exceeds 0.1 and the *I*^2^ value is less than 50%, and the fixed-effects model will be used. While studies will be considered to have significant statistic heterogeneity if the *P* value is less than .1 or the *I*^2^ value exceeds 50%, and subgroup analysis will be used to explore the possible cause. And the random-effects model will be applied if the heterogeneity is still important.

#### 2.5.8 Assessment of reporting biases

Funnel plots will be used to access the reporting biases if there are over 10 trials included in the meta-analysis.

#### 2.5.9 Data synthesis

Review Manager Software (RevMan) V.53 will be used for data synthesis. The random-effects model will be used if the *I*^2^ value is no less than 50%. The fixed-effects model will be used if the heterogeneity tests show little statistical heterogeneity. If there is meaningful heterogeneity that cannot be explained by any assessment, meta-analysis will not be performed.

#### 2.5.10 Subgroup analysis

There is no presubgroup plan. Subgroup analysis will be conducted if data are available. Factors such as different types of control interventions and different outcomes will be considered.

#### 2.5.11 Sensitivity analysis

Sensitivity analysis will be conducted to test the robustness of the review conclusions if possible. The impacts of sample size, study design, methodological quality, and missing data will be evaluated.

#### 2.5.12 Grading the quality of evidence

The Grading of Recommendations Assessment approach will be used to judge the quality of the evidence for all outcomes [14]. Risk of bias, heterogeneity, indirectness, imprecision and publication bias will be assessed. The assessments will be classified into 4 levels: high, moderate, low, or very low.

#### 2.5.13 Ethics and dissemination

This protocol will not evaluate individual patient information or affect patient rights and therefore does not require ethical approval. Results from this review will be disseminated through peer-reviewed journals and conference reports.

## 3 Discussion

This systematic review will assess the effectiveness and safety of acupuncture for chronic constipation in patients with diabetes mellitus. There are 4 sections in the review: identification, study inclusion, data extraction and data synthesis. This review will help the doctors to choose acupuncture as an alternative treatment for chronic constipation in patients with diabetes mellitus, and offer the patients more options to relieve their symptoms.

## Author contributions

SFC, QY, and SHX mainly contributed to this manuscript and joint first authors. GJZ obtained funding. SFC drafted the protocol. QY make the search strategy. SHX will obtain copies of the studies and screen the studies to be included. Data extraction from the studies will be done by QL. SFC will put the data into RevMan. Analyses will be conducted by SHX. SFC will draft the final review and GJZ will update the review. GJZ will act as an arbiter in the study selection stage. All authors have read and approved the final manuscript.

**Data curation:** Sufen Cui.

**Methodology:** Qiu Yang.

**Software:** Suhua Xie.

**Validation:** Qian Liu.

**Writing – review & editing:** Guangju Zhou.
